# Fully Bio-Based Elastomer Nanocomposites Comprising Polyfarnesene Reinforced with Plasma-Modified Cellulose Nanocrystals

**DOI:** 10.3390/polym13162810

**Published:** 2021-08-21

**Authors:** Ilse Magaña, Dimitrios Georgouvelas, Rishab Handa, María Guadalupe Neira Velázquez, Héctor Ricardo López González, Francisco Javier Enríquez Medrano, Ramón Díaz de León, Luis Valencia

**Affiliations:** 1 Research Center for Applied Chemistry, Blvd Enrique Reyna 140, San José de los Cerritos, Saltillo 25294, Mexico; ilsma.rivera58@gmail.com (I.M.); guadalupe.neira@ciqa.edu.mx (M.G.N.V.); ricardo.lopez@ciqa.edu.mx (H.R.L.G.); javier.enriquez@ciqa.edu.mx (F.J.E.M.); 2 Division of Materials and Environmental Chemistry, Stockholm University, Frescativägen 8, 10691 Stockholm, Sweden; dimitrios.georgouvelas@mmk.su.se; 3 Experimental Physics, Saarland University, 66123 Saarbrücken, Germany; rishabhanda93@gmail.com; 4 Biofiber Tech Sweden AB, Birgen Jarksgatan 57 C, 11356 Stockholm, Sweden

**Keywords:** cellulose nanocrystals, bio-based, elastomer nanocomposites, *trans*-β-farnesene, plasma-induced polymerization, surface modification

## Abstract

This article proposes a process to prepare fully bio-based elastomer nanocomposites based on polyfarnesene and cellulose nanocrystals (CNC). To improve the compatibility of cellulose with the hydrophobic matrix of polyfarnesene, the surface of CNC was modified via plasma-induced polymerization, at different powers of the plasma generator, using a *trans*-β-farnesene monomer in the plasma reactor. The characteristic features of plasma surface-modified CNC have been corroborated by spectroscopic (XPS) and microscopic (AFM) analyses. Moreover, the cellulose nanocrystals modified at 150 W have been selected to reinforce polyfarnesene-based nanocomposites, synthesized via an in-situ coordination polymerization using a neodymium-based catalytic system. The effect of the different loading content of nanocrystals on the polymerization behavior, as well as on the rheological aspects, was evaluated. The increase in the storage modulus with the incorporation of superficially modified nanocrystals was demonstrated by rheological measurements and these materials exhibited better properties than those containing pristine cellulose nanocrystals. Moreover, we elucidate that the viscoelastic moduli of the elastomer nanocomposites are aligned with power–law model systems with characteristic relaxation time scales similar to commercial nanocomposites, also implying tunable mechanical properties. In this foreground, our findings have important implications in the development of fully bio-based nanocomposites in close competition with the commercial stock, thereby producing alternatives in favor of sustainable materials.

## 1. Introduction

During the last century, the use of polymeric materials has diversified into different aspects of our lives, with uses ranging from consumer products to industrial applications. However, limited fossil resources make their growing manufacturing unsustainable for future generations. Therefore, one of the main actions to overcome this problem consists of the use of biopolymers, derived from renewable natural resources, to produce materials with competitive performance. In this context, the implementation of terpenes as precursors of sustainable elastomers appears as a prominent alternative. Terpenes are natural unsaturated hydrocarbons produced by plants, which can be considered renewable raw materials for obtaining bio-based chemicals and polymers. In this sense, simple acyclic monoterpenes, particularly β-myrcene, ocimene, and *trans*-β-farnesene, provide similar chemistry to the already known unsaturated hydrocarbons derived from oil and natural gas, highlighting among them 1,3- butadiene and isoprene [1]. *Trans*-β-farnesene is a member of a group of sesquiterpene isomers found in apple peel, citrus fruits, and essential plant oils, although it is also released by aphids. Currently, *trans*-β-farnesene is commercially produced by fermenting cane sugar using a genetically modified yeast [2]. This biological monomer has already been successfully polymerized by free radicals [3] and anionic [4] polymerization techniques; however, only the polymerization by coordination provides polyfarnesene (PF) with a high stereospecificity [5,6], which, depending on the application, generally results in a better performance. The applications of elastomers at an industrial level commonly demands reinforcements linked to the use of different types of fillers to improve the materials’ final properties, such as mechanical performance. In this context, nanocellulose (NC), specifically cellulose nanocrystals (CNC), appear to be one key sustainable alternative due to their excellent mechanical properties, as well as their biodegradability and versatility of surface modification.

There are different ways of preparing nanocomposites, for instance via compounding using a twin-screw extruder (TSE) or via in situ polymerization. The latter involves carrying out the polymerization in the presence of the nanoparticles. According to the literature, it is generally known that in situ polymerization in the presence of fillers has distinct advantages over other nanocomposite synthesis procedures, appearing to be more suited in generating superior nanoparticle dispersion, which should have a stronger impact on the attributes achieved [7]. Until now, the in-situ polymerization technique has been reported to obtain polymeric nanocomposites with cellulose nanocrystals using ring-opening [8], radical [9], and atom transfer radical [10] polymerization as pathways; however, until now it has not been reported through coordination polymerization. One of the main reasons that in-situ polymerization has not been tested using this technique may be because polymerization by coordination is a reaction sensitive to impurities as well as electrodonate groups, such as hydroxyls, which are present on the surface of cellulose nanocrystals.

Although the reinforcement capabilities of CNC have been widely reported for multiple polymer matrices [11,12,13,14,15], their compatibility with apolar polymer matrices is restricted by their hydrophilic nature. However, the abundance of hydroxyl groups on the surface of CNC provides the versatility of functionalization to make them hydrophobic, and thus more compatible with different polymers. Recently, we have demonstrated that plasma-induced polymerization appears to be an efficient strategy to surface-modify CNCs for polymer reinforcement [16]. Moreover, this technique is eco-friendly, inexpensive, and does not affect the general features of the substrate [17,18,19,20,21,22].

**Figure 1:**
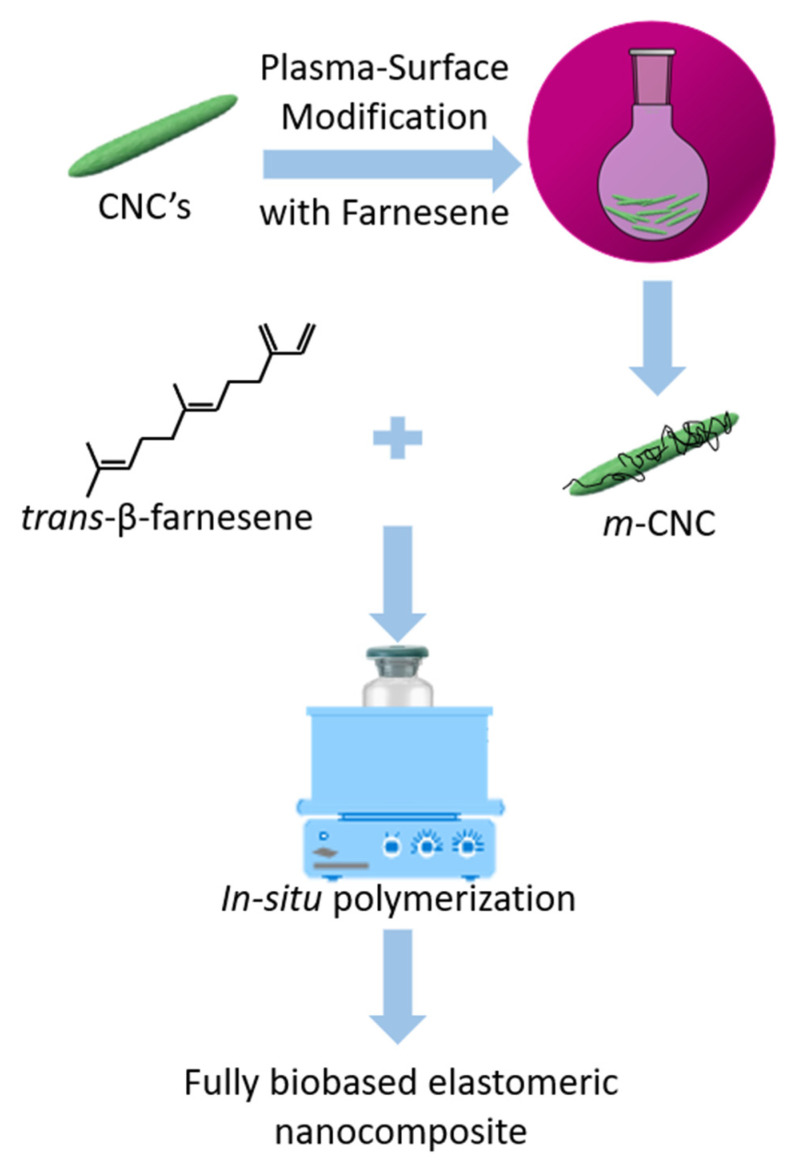
Schematic illustration of the process to obtain fully biobased elastomeric nanocomposites.

In this work, we report the preparation of fully bio-based elastomer nanocomposites based on polyfarnesene reinforced with plasma-modified cellulose nanocrystals at different loading contents. The nanocomposites were prepared via in-situ polymerization, using coordination polymerization by means of a neodymium-based catalyst. The effect of CNC on reaction kinetics was elucidated, as well as over the macro and microstructure of the resultant polymers. Moreover, a rheological characterization revealed the mechanical performance of the material, demonstrating the potential to enhance the elastic properties of fully biobased nanocomposites. A schematic diagram of the whole process is shown in Figure 1. The samples obtained from this work can be crosslinked/vulcanized to test their use and performance in the various areas of rubber application. Until now, no farnesene-based elastomer with cellulose nanocrystals as a reinforcement has been reported. It is mainly oriented towards creating new alternatives in the rubber industry for its application as totally biobased bio-rubbers for the new generation of green tires.

## 2. Materials and Methods

### 2.1. Materials

Cellulose nanocrystals were supplied by Celluforce (Montreal, QC, Canada). Cyclohexane (reagent grade, purity 99%) was supplied by Sigma Aldrich, and *trans*-β-farnesene (reagent grade, purity > 99%) was provided by amyris (Emeryville, CA, USA). The cyclohexane was washed with sulfuric acid and then refluxed in two stages, the first with lithium aluminum hydride and the second with metallic sodium. The *trans*-β-farnesene was washed two times with a 1 M sodium hydroxide solution, then dried over sodium sulfate and finally distilled under reflux and metallic sodium. Diisobutyl aluminum hydride (DIBAH, 1 M in hexane, reagent grade) and dimethyl dichlorosilane (Me_2_SiCl_2_, purity > 99.5%) were purchased from Sigma Aldrich. The latter was used in solution with purified cyclohexane at a concentration of 0.22 M. Neodymium versatate (NdV_3_-40, 0.54 M in hexane, reagent grade) was provided by Solvay (Brussels, Belgium).

### 2.2. Methods

#### 2.2.1. Surface Modification of CNCs

Plasma-induced polymerization was carried out based on the experimental procedures described by Neira et al., [23] using a custom-made plasma reactor. A schematic representation of the setup is shown in our previous work [16]. For the process, 2 g of CNC were introduced to the reaction chamber, which was sealed and then subjected to a vacuum until reaching a pressure of 1.5 × 10^−1^ mbar. Once the pressure was stabilized, trans-β-farnesene (25 mL of monomer was added into a flask connected to the Schlenk line. After three reactions, a loss of approximately 4 mL was observed) was left to flow through for 20 min so that the chamber was saturated with the monomer. After this time, and ensuring a stable pressure in the system, the plasma was turned on at a determined power (70, 100, or 150 W). The treatment time was 60 min. Upon completion, the plasma generator was turned off and the system was depressurized. The modified nanocrystals (hereinafter referred to as *m*-CNC) were removed from the reaction chamber and stored in glass vials for later characterization. The samples were named according to plasma power (W) used during the process as (*m*-CNC_70_), (*m*-CNC_100_) and (*m*-CNC_150_).

#### 2.2.2. In-Situ Polymerizations

Vacuum-dried CNC were exposed to nitrogen atmosphere in a 100 mL vial by using a glove box. Then, 80 mL of cyclohexane and 15 mL of *trans*-β-farnesene were added and dispersed by sonication (40 kHz sonication frequency, 40 min). Then, the *m*-CNC suspension (the concentrations by weight of nanocrystals with respect to the monomer used were 0.0, 0.5, 1.5, 3.0 and 5.0%) was incorporated into the reaction system, comprising (pre-prepared in a glove box) NdV_3_, DIBAH and Me_2_SiCl_2_ at 70 °C and left for 30 min under vigorous stirring, to carry out the polymerization reaction. Sufficient reaction time was given to obtain a conversion close to 100%. The polymerization monitoring was carried out by taking samples of the reaction mixture every 5 min to evaluate gravimetrically the evolution of the polymer yield. After 30 min the reaction was terminated with the addition of antioxidants in methanol at 0.5 wt%. The nanocomposite was separated by precipitation in acidified methanol. Finally, it was vacuum-dried at 50 °C prior to storage.

### 2.3. Characterization

#### 2.3.1. Surface Modification of CNC

The chemical composition of the m-CNC was analyzed by ATR-Fourier Transform Infrared Spectroscopy (FTIR) using a Thermo Scientific Nicolet iS5 FT-IR (Thermofisher Scientific, Waltham, MA, USA) spectrometer in a range of 400 to 4000 cm^−1^, the spectra were obtained at 32 scans and a resolution of 4 cm^−1^. The crystallinity was determined by X-ray Diffraction (XRD) using Bruker Eco D8 Advance (Billerica, MA, USA). The crystallinity index (CrI) was calculated using the equation of the Segal method [24]. The thermal stability was studied by Thermogravimetric Analysis (TGA) with a TA-Q500 thermo-analyzer (TA Instruments, New Castle, DE, USA), heating the samples from room temperature to 600 °C at a constant rate of 10 °C/min under a nitrogen flow of 50 mL/min. The surface chemical composition was analyzed by X-ray Photoelectron Spectroscopy (XPS), using a Riber LDM-32 (ISA-Riber, Ruel-Malmaison, France) spectrophotometer, with an aluminum anode operating at 150 W with a step of 20 eV for the individual photoelectron lines. For the deconvolution of the high-resolution spectrum of C 1s, an adjustment was performed using a Shirley background subtraction and a series of Voight peaks. The morphology and topography of the samples were revealed by Atomic Force Microscopy (AFM) with a Nanoscope V system (Veeco Instruments, Santa Barbara, CA, USA), using the Tapping mode in the air at room temperature.

#### 2.3.2. Elastomeric Nanocomposites

The molecular weight characteristics of the polymers were determined by Size Exclusion Chromatography (SEC) using PL-GPC 50 equipment (Agilent Technologies, Santa Clara, CA, USA), configured with a 5 µm mixed type column at a pressure of 4.54 MPa and a refractive index detector calibrated with polystyrene standards. The samples were prepared by dispersing the PF (industrial grade, purity > 90% from Quifersa (Saltillo, Mexico)) in toluene and centrifuged to separate the nanoparticles, then precipitated in methanol (industrial grade, purity > 99%, provided by Quifersa). The synthesized materials were dissolved in THF (HPLC grade supplied by Sigma Aldrich) in a 1:1 ratio; that is, 1 mg of sample in 1 mL of THF, then they were filtered through a filter with a pore size of 0.2 µm. The microstructure of the PF was studied by ^1^H and ^13^C Nuclear Magnetic Resonance (NMR) using a 500 MHz Bruker Ultrashield Plus spectrophotometer. The samples were also filtered to remove the nanocrystals and then dissolve the sample in deuterated chloroform (CDCl_3_). The glass transition temperature (T_g_) was analyzed by Differential Scanning Calorimetry (DSC) analysis with a Discovery 2500 equipment (TA Instruments, New Castle, DE, USA) using a heating/cooling rate of 10 °C/min, around 10 mg of the sample was used. A first heating cycle from −90 to 30 °C was performed to remove the thermal history of the polymer, a second cycle corresponded to the cooling of the sample from 30 to −90 °C, finally another heating cycle was carried out from −90 to 30 °C. The thermal stability of the nanocomposite was determined by TGA using Q-500 equipment (TA Instruments New Castle, DE, USA), with a heating ramp of 10 °C/min from room temperature to 600 °C under a nitrogen flow of 50 mL/min, about 10 to 30 mg of sample was used. The rheological properties were studied with a Q300 oscillating rheometer (Anton Parr, Graz, Austria), with a plate/plate configuration with a diameter of 25 mm with 1 mm gap between plates at room temperature, using a constant strain of 5% (in the linear viscoelastic range) with a frequency sweep of 0.01 to 300 rad/s.

## 3. Results and Discussion

### 3.1. Modified Cellulose Nanocrystals

**Figure 2:**
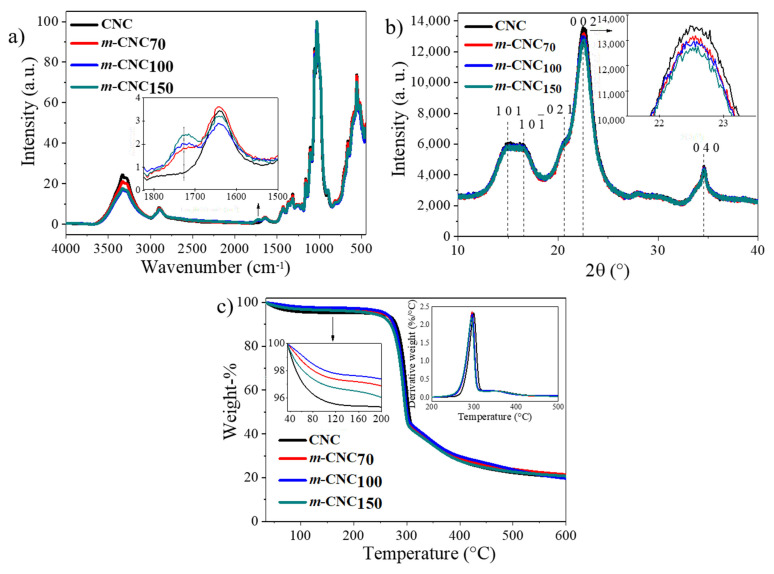
Characterization of the untreated and modified-cellulose nanocrystals. (**a**) FTIR spectra; (**b**) XRD; (**c**) TGA thermogram.

To improve the compatibility of CNC with the hydrophobic matrix of polyfarnesene, plasma-induced polymerization was herein used to modify the cellulosic surface using farnesene monomers in the plasma reactor, at different powers (70, 100, and 150 W). The samples were named according to plasma power (W) used during the process as (*m*-CNC_70_), (*m*-CNC_100_), and (*m*-CNC_150_). The successful surface-modification of the nanocrystals was first proved by FTIR spectroscopy (Figure 2a). As can be observed, the spectra displayed the signals corresponding to the cellulosic structure, as expected, including the stretching vibration of the C-H bonds at 2900–2800 cm^−1^ and the asymmetric stretching vibrations of C-O-C at 1161 cm^−1^ present in the pyranose ring [25,26,27]. No significant changes in the FTIR spectra could be observed upon modification, due to the small peaks corresponding to the double bonds in the structure of the polyterpenes. Nevertheless, the appearance of a signal at 1700 cm^−1^ upon surface-modification was evident, which could correspond to oxidized species on the surface of the nanocrystals upon the surface-modification. A slight decrease in the CrI of the *m*-CNC was demonstrated via XRD (Figure 2b), the values of which are as follows: 72.3, 71.9, 70.7, and 69.9% for CNC, *m*-CNC_70_, *m*-CNC_100_, and *m*-CNC_150_, respectively. This phenomenon does not suggest that the CNCs were intrinsically less crystalline, but rather elucidates the presence of the deposited polymer by plasma-induced polymerization (which has an amorphous nature). The amorphous content in the samples increased as a function of the plasma power, suggesting a greater extent of modification.

The thermal behavior of the nanocrystals was studied by TGA (Figure 2c). A first weight loss near 100 °C can be observed in all samples, attributed to the moisture-loss adsorbed by the sample. A lower moisture loss was observed for the *m*-CNC, which suggests that they have a more hydrophobic character, due to the deposition of the hydrophobic polymer coating, thus suggesting better compatibility with the polymeric matrix. For all samples, a second degradation stage could be noted near 250 °C, corresponding to the breaking of the cellulosic glycosidic bonds. Finally, a third stage can be observed around 300 °C, corresponding to the second stage in the degradation of cellulose [28]. The thermograms of *m*-CNC show slight differences compared to that of CNC, indicating that there is no impact on the thermal stability of the nanocrystals upon the surface modification. The amount of coated polymer is too little to be quantified through this method.

**Figure 3:**
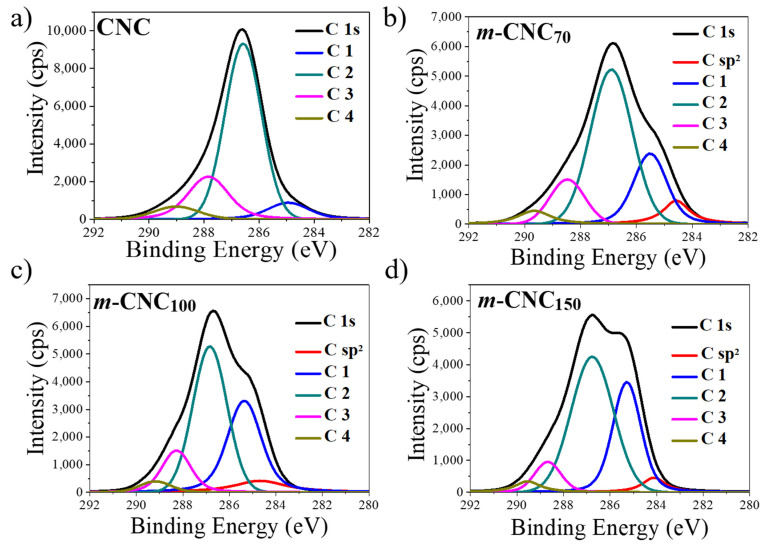
Peak-fitted high-resolution C 1s XPS spectra of the unmodified (**a**) and modified (**b**–**d**) cellulose nanocrystals.

The chemical composition of the cellulose nanocrystals, before and after modification, was analyzed via XPS, by comparing the deconvoluted C 1s peak located at a binding energy of 286 eV (see Figure 3a) (The survey spectra is shown in Figure S1). The integral area of the fitted peaks from the deconvolution is shown in Table 1. For cellulose, the C 1s signal can be fitted into four populations (Voigt distributions), corresponding to the different bonding to carbon. Carbon atoms bonded to hydrogen atoms or other carbon atoms correspond to the C 1 type (285 eV). C 2 type (287 eV) is related to carbon atoms bonded to an oxygen atom (C-O, C-OH). Carbon atoms bonded to a carbonyl, or two non-carbonyl oxygen atoms (C=O, O-C-O) corresponds to C 3 type (288 eV), and the class of C 4 (289 eV) associates with carbon atoms bonded to a carbonyl and non-carbonyl oxygen atom (O=CO) [16,29,30,31,32,33]. All the distinctive signals could be observed in all types of nanocrystals; however, the *m*-CNC exhibits an additional signal at a binding energy of 284 eV, which is attributed to the sp^2^ bond of carbon, from the double bonds in the backbone and side chains of PF deposited on the surface of the *m*-CNC [16].

Furthermore, the carbon to oxygen ratio also appears to increase upon the surface-modification, due to the aliphatic nature of the deposited polymer during the modification (Table 1). Moreover, it appears that the power used in the plasma modification significantly increments the C 1 type carbons, indicating the greater content of aliphatic polymer deposited upon surface modification [16].

**Figure 4:**
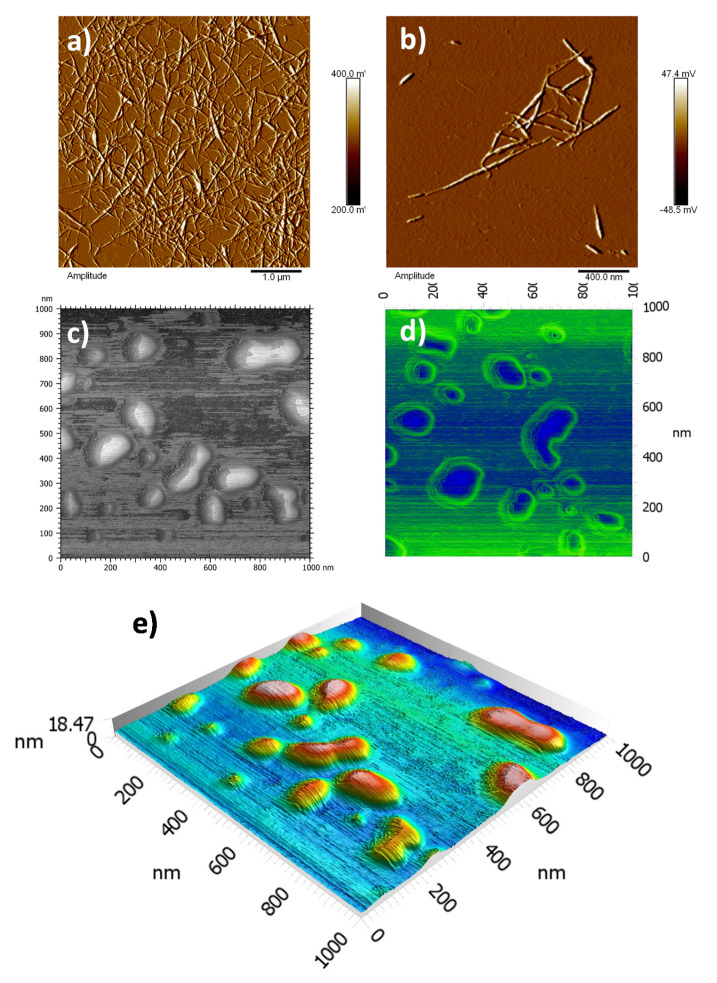
Micrographs obtained by AFM of (**a**,**b**) unmodified CNC, and (**c**) modified CNC. (**d**) Phase mode and (**e**) 3D micrographs of modified cellulose nanocrystals.

The topography of the cellulose nanocrystals was studied by AFM (Figure 4a,b). The pristine CNC exhibits a typical rod-like structure with lengths fluctuating between 120–200 nm and diameters of about 6–8 nm. On the other hand, the *m*-CNC (Figure 4c,e) were found in the diameter range 7–25 nm and lengths between 150–230 nm, which is attributed to the deposition of polymeric material on the surface. The phase micrograph (Figure 4d), furthermore, revealed the deposited polymer on the *m*-CNC with good contrast due to their difference in composition. It is noteworthy that the presence of aggregates was also found (see Figure S2), with significantly larger sizes.

### 3.2. Synthesis of Nanocomposites

**Figure 5:**
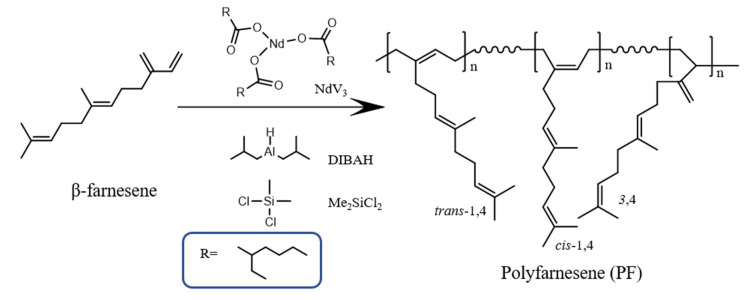
Scheme of the polymerization of *trans*-β-farnesene using the catalytic system comprising NdV_3_ (catalyst), DIBAH (co-catalyst), and Me_2_SiCl_2_ (halide donor).

To prove the enhancement in polymer compatibility upon the plasma-surface modification, the pristine CNC (as a control sample) modified at 150 W (*m*-CNC_150_) were tested as reinforcement additives for PF matrix at different concentrations and they are named as PF/CNC (samples from PF-1 to PF-4) and PF/*m*-CNC_150_ (samples from PF-5 to PF-8), respectively, where pure PF was used as a reference to the nanocomposites. The resultant nanocomposite was prepared via in-situ polymerization (polymerizing the monomer in the presence of the nanofiller), elucidating the influence of the nanocrystals over the polymerization behavior and polymers’ final properties. The synthesis was carried out via coordination polymerization using the catalytic system that comprises NdV_3_ (catalyst), DIBAH (co-catalyst), and Me_2_SiCl_2_ (halide donor) (see Figure 5). The use of this type of polymerization offers the advantage to control the microstructure of polymers, especially polydienes, which is one of the essential and required characteristics to consider at an industrial level to produce high-performance rubbers. However, the employed catalytic system is sensitive to deactivation because of impurities such as water. NdV_3_ is one of the most reported rare earth metal-based catalysts and several studies have already been published about its application to polymerize terpenes with good control of macro- and microstructures [5,6,34,35,36], including the in-situ preparation of nanocomposites including graphene oxide [37]. The mechanism of a conventional coordination reaction comprises the steps of catalyst activation, initiation, propagation, and chain transfer [38,39]. However, under certain molar ratios, the alkylaluminum (co-catalyst) acts as a transfer agent; therefore, reversible chain transfer reactions occur, entering a coordinative chain transfer polymerization (CCTP) regime. This allows control of the dispersity of the thereof resultant polymers [40,41].

**Figure 6:**
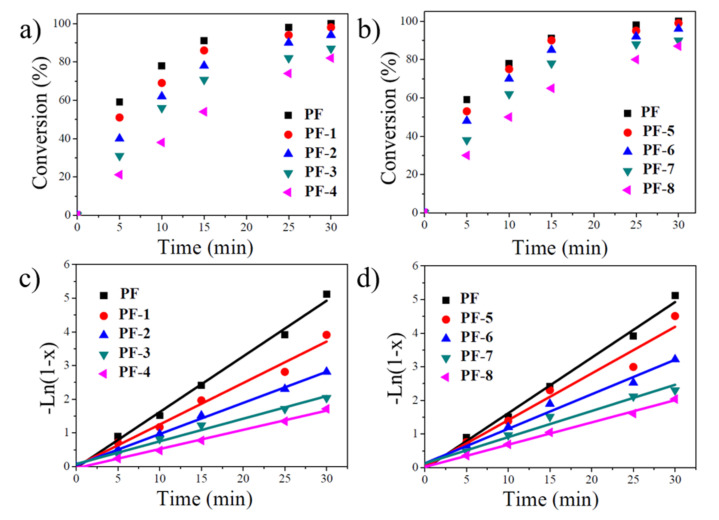
Polymerization behavior of the in-situ polymerizations of farnesene and modified/unmodified cellulose nanocrystals: (**a**,**b**) Evolution of conversion with time, (**c**,**d**) linear form of the conversion-time plots.

The conversion-% evolution as a function of time for the farnesene polymerization reactions is shown in Figure 6a,b where it is observed how the conversion is affected when incorporating both CNC and *m*-CNC_150_, resulting in a lower yield as a function of nanocrystal content. The kinetics of the polymerization reactions were studied by analyzing the conversion-time plots (see Figure 6c,d). The straight lines, which could be described as a first-order kinetics relation with respect to the monomer, indicating the living behavior of the polymerizations [38,42].

The calculated apparent kinetic rate constant (k), as well as the catalytic activity and the reaction conversion, are presented in Table 2. These kinetic parameters decreased by 66% and 18%, respectively, when adding 5 wt% of CNC compared to PF. Regarding the in-situ polymerization with 5 wt% *m*-CNC_150_ (PF-8), the polymerization rate decreases by 60% and the catalytic activity by 13%. These parameters are mostly affected by adding a concentration of the nano-filler equal to or greater than 1.5 wt%. Such a decrease in conversion-% can be attributed to the sensitivity towards deactivation by impurities such as moisture or oxygenated compounds by the coordination complexes generated from the Ziegler Natta-based neodymium catalyst used. The presence of double bonds on the *m*-CNC_150_ surface could react in the polymerization, presumably deactivating partially the catalytic species and therefore affecting the kinetics of the reaction. Additionally, the hydroxyl groups present on the surface of CNC also have the potential to interact or react with the co-activators of the catalytic system, which can be present individually in the polymerization. This implies that the -OH groups of pristine, as well as of the modified CNC, when reacting with the aluminum present in the DIBAH (required to achieve the CCTP regime), can cause the [Al]/[Nd] ratio to be shifted towards lower values, resulting in fewer active catalytic species, which explains the significant increase in molecular weight and dispersity (M_w_ and Ð) of the synthesized polyfarnesenes (Table 2) in the presence of CNC and *m*-CNC_150_.

Broadening of the molecular weight distributions (MWD) and multimodality was also observed upon the incorporation of CNC and *m*-CNC_150_ (see Figure S3). This supports the hypothesis of partial deactivation of the catalytic system and consumption of DIBAH, compromising the CCTP regime by breaking the equilibrium in the reversible transfer reactions. Consequently, this favors termination via irreversible transfer, preventing the controlled growth of the polymeric chains [6,40,43,44]. In this case, the appearance of different populations is attributed to the action of at least two active species with different reactivity. Furthermore, regardless of the nanocrystals (modified and unmodified) used, the selectivity of the catalyst system was not affected, as corroborated by the negligible change in *cis*-1,4 percentage (see Table 2).

### 3.3. Rheological Properties of the Elastomer Nanocomposites

**Figure 7:**
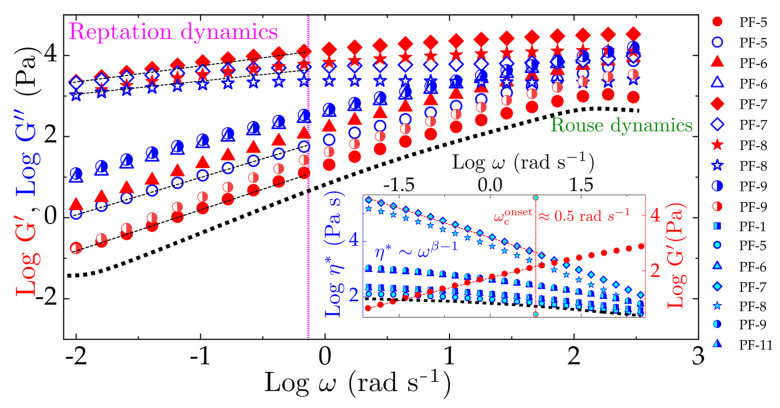
Viscoelastic moduli (G’ and G’’) regime for pure PF (dotted line, in black) and PF nanocomposites incorporated with CNC and *m*-CNC_150_ in a frequency sweep protocol. Inset: Viscosity is depicted as a function of angular frequency, where red circles (G’) serves as a reference to highlights a crossover point between elasticity and viscosity indicating an onset ω_c_ = 0.5 rad * s^−1^.

Despite the staggering progress made in understanding the mechanical response of nanocomposites, as delineated by extensive literature [45,46,47,48,49,50,51,52], it still lacks a theoretical framework to determine their governing dynamics and thus offers room for improvement. Microstructural changes in sheared polymer composites strongly affect their viscoelastic behavior, causing rheological tests to be quite sensitive. Figure 7 summarizes the storage (G’) and loss modulus (G’’) obtained in a frequency sweep tests for polyfarnesene nanocomposites reinforced with pristine cellulose nanocrystals (PF/CNC, half-filled symbols) and modified cellulose nanocrystals (PF/*m*-CNC_150_, filled symbols). The protocol of the test was carried out following the some works in the literature [53,54,55], which allowed us to identify and represent a rough picture of important chain dynamics as corroborated by the fits of viscoelastic moduli.

In the purview of tube models, at sufficiently small frequencies and length scales larger than the entanglement strand size, the topological constraints (entanglements) define the curvilinear motion of a polymer in the vicinity of its tube [53]. Such behavior of a polymer is characterized by the longest stress relaxation time (reptation time $\tau_{d}$), with moduli scaling as, $\;G\prime \sim \omega^{2}$ and $\;G\prime \prime \sim \omega^{1}$, where $\omega \leq 1/\tau_{d}$ [53], corresponding to the dashed fits in Figure 7, respectively. However, as frequency sweeps to higher values, allowing length scales to be smaller than topological constraints or strand size, entanglements and hydrodynamic drag can be unscreened and neglected, and the diffusion of polymer chains are characterized by a Rouse friction coefficient, determined by Rouse time $\tau_{R}$, with $\;G\prime \left(\omega \right)=\;G\prime \prime \left(\omega \right)\sim \omega^{1/2}$, $\forall \;\omega >1/\tau_{e},$ where $\tau_{e}\equiv 2\tau_{R}$ [53,55]. In this regard, Figure 7 considerably captures the aforesaid stress relaxation dynamics with $\tau_{R}\approx 0.04\;s$ for pure PF and $\tau_{R}\approx 9s$ for polyfarnesene with the highest content of *m*-CNC_150_ (PF-8) estimated at 298 K using an equation of the form $\;\tau_{R}={\left(aM/1.111bRT\right)}^{2}$ adopted from the work [54], where a is obtained by fitting the storage modulus as $\;G\prime =a\omega^{1/2}$, M is the molecular weight, and *b* is the mass of polymer per unit volume. Table 2 lists the molecular weight distribution and additional parameters. Having the stress relaxation of nanocomposites approximated with an agreeable scaling exponent [54,56,57,58], we find the order of magnitude difference in $\tau_{R}$ to be related to the microstructural features of a CNC-embedded PF composite. Adding CNC and *m*-CNC_150_ to the PF increased the $M_{w}$ in a power-law fashion (see Table 2), corroborating to a higher $\tau_{R}$, as the entanglement molecular weight and the polydispersity (Ð) will extend the relaxation time span [53]. In addition, high deformation stretches the entangled chains, causing strain overshoot before steady-state rheology is reached. A complex viscosity for pure PF and PF/CNC composites has been summarized in Figure 7 inset, along with a reference to  G′ (in red), to find the onset of phase transition determined by the critical frequency $\omega_{c}$ at a crossover of $\eta^{*}$ and G′ [58]. The pseudo-plastic plateau for $\eta^{*}$ appears to be higher for a low content of CNC (0.5–1.5 wt%, regardless of pristine or modified attributes) in the PF matrix than those with a higher content and in particular, ones reinforced with *m*-CNC_150_ (filled symbols: stars and diamonds see the inset of Figure 7). Shear-thinning has been exhibited in all the samples, although it increases with the content of CNC in PF matrix, with PF/CNC being slower than PF/*m*-CNC_150_. The significance of molecular weight and CNC content in PF is acknowledged by the fact that, in contrast to the preceding PF nanocomposites with pristine CNC and *m*-CNC_150_, another set of nanocomposites were synthesized while keeping their molecular weights under control in reference to the pure PF to elucidate the influence of various loading contents of modified and unmodified cellulose nanocrystals. The synthesis of PF-CNC and PF-*m*-CNC_150_ was carried out by increasing the concentration of the catalyst to compensate for the deactivation of active species for polymerization, allowing control over the molecular weight to obtain polymeric matrices for the nanocomposites with similar molecular weights (Table 3). Moreover, no direct relationship between the concentration of the catalytic system and the content of nanofillers was found.

Rescaling the viscosity with a power-law of the form $\eta^{*}\sim \omega^{\beta -1}$ reveals for PF/CNC as $\eta^{*}\sim \omega^{1}$ and PF/*m*-CNC_150_ as $\eta^{*}\sim \omega^{1/3}$. These exponents indicate that adding *m*-CNC_150_ has a stronger influence on the frequency dependence, thus offering to control the microstructural changes and relaxation dynamics by adjusting the concentration of CNC. Besides, zero-shear viscosity $\eta_{0}^{*}$ was estimated via the Carreau-Yasuda model, $\eta^{*}=\eta_{0}^{*}{\left[1+{\left(\lambda \omega \right)}^{\alpha }\right]}^{\left(\beta -1\right)/\alpha }$, where λ is the ratio of $\eta_{0}^{*}$ to the stress at a crossover of Newtonian to non-Newtonian response, α is a transition adjusting exponent, and β is the power-law exponent [56,58].

**Figure 8:**
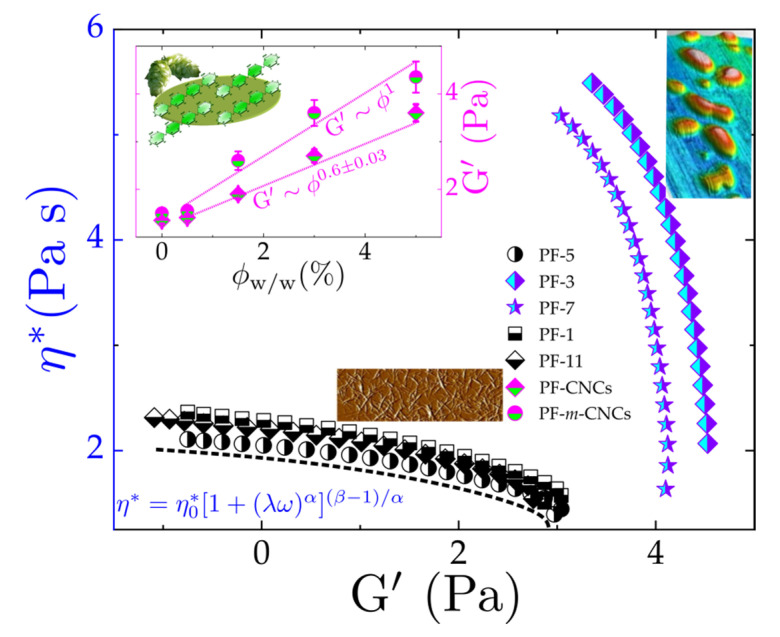
Viscosity depicted at the corresponding elastic modulus, indicating percolation or agglomeration of CNC in contrast to pure PF (dashed line, in black), as illustrated by the images. Inset: Concentration dependence of the terminal modulus (G’ at $\omega_{c}=0.5\;{\mathrm{rad}\;s}^{-1}$) diamonds represent PF-CNC; circles represent PF-*m*-CNC.

**Figure 9:**
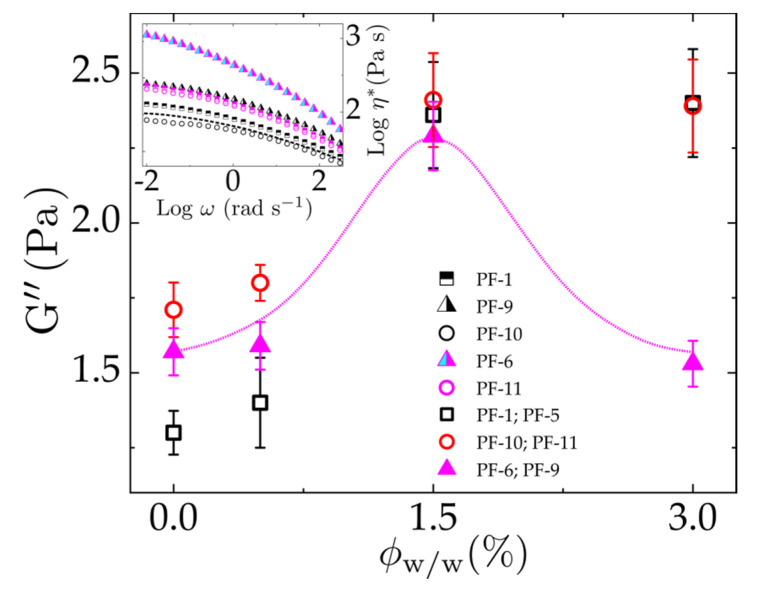
Loss modulus depicted as a function of concentration, confirming the percolation threshold at a CNC concentration of 0.5%. Inset shows the viscosity comparison between the modified and initial set of samples as described in Table 3 where PF in a dashed line serves as a reference.

The abrupt increase in $G_{\omega_{c}}^{\prime }$ $\phi_{w/w}\sim 1.5\%$ (chosen at $\omega_{c}$) alludes to the vanishing reptation dynamics (see Figure 8). Since increasing the concentration of cellulose nanocrystals (modified and unmodified) in PF facilitates the CNC network formation, these emerging structural networks thus hinder the stress relaxation and dissipation dynamics as they begin to percolate. Besides, the fits shown on the inset of Figure 8 follows the fractal-like model system $G’\sim \phi^{n}$, which is found to be larger for PF-*m*-CNC_150_ ($G’\sim \phi^{1}$) than PF/CNC ($G’\sim \phi^{2/3}$) nanocomposites. On this account, we represent G’’ (viscous dissipation) as a function of normalized *m*-CNC concentration in Figure 9. A fit of Gaussian-Lorentzian function clearly reveals a percolation threshold at $\phi_{w/w}\sim 1.5\%$**** as illustrated in Figure 9, which indicates the concentration where the particles begin to interconnect and form a network within the polymeric matrix. This is determined rheologically by the liquid–solid transition in the viscoelastic behavior [57,58]. Nevertheless, samples with a higher content of modified CNC in PF and the older set with uncontrolled M_n_ exhibited a saturation peak following a power law; hence, this implies a coexistence of partial percolation and agglomeration. The inset is a reference to the implication of percolating *m*-CNC on the viscosity of the material, comparing PF/*m*-CNC to PF/*m*-CNC_150_.

## 4. Conclusions

We have confirmed that plasma modification is a practical and successful technique for the surface modification of cellulose nanocrystals without altering their intrinsic properties. Besides this, hints were obtained through a relationship between the power used in the plasma modification with the content of polymer deposited on the surface. Furthermore, we demonstrated the possibility of preparing elastomeric nanocomposites (based on polyfarnesene) with CNC and *m*-CNC_150_ via in-situ coordination polymerization using a neodymium-based catalyst. The resultant polymers exhibited good control in microstructure (high 1,4-*cis*), and their conversion time plots could be described as first-order kinetic relations with respect to the monomer, indicating the living behavior of the polymerizations. It was also determined that under the studied concentrations, the content of nanocrystals equal to or greater than 1.5 wt% partially deactivated the catalytic system, affecting the kinetic parameters of the polymerization reaction. On the other hand, we demonstrated the increase in storage modulus upon the incorporation of surface-modified nanocrystals, exhibiting greater properties to pristine CNC. We identified a percolation threshold at 1.5 wt-%, where a maximum in G´ is evident. The viscoelastic moduli of nanocomposites are aligned with power-law model systems with characteristic relaxation time scales like commercial nanocomposites, thus implying tunable mechanical properties. This work elucidates a 100% biobased elastomeric material prepared through in-situ polymerization using the coordination polymerization route.

## Figures and Tables

**Table 1:** Surface composition of untreated and modified CNC calculated by XPS and the C 1s high-resolution data.

Sample	C/O	C 1 (%)	C 2 (%)	C 3 (%)	C 4 (%)	C sp^2^ (%)
CNC	0.47	8.2	70.3	15.3	6.1	-
***m*** **-** $CNC_{70}$	0.54	19.6	54.5	12.5	5.0	8.4
***m*** **-** $CNC_{100}$	0.62	26.4	53.5	9.3	3.2	7.5
***m*** **-** $CNC_{150}$	0.78	36.2	46.9	8.1	4.2	4.5

**Table 2:** Effect of the incorporation of CNC and *m*-CNC in the polymerization of farnesene, and the macro- and microstructure of the resultant polyfarnesenes.

Run	Additive	wt%	Yield ^a^	*K* ^b^	A ^c^	$M_{w}$(kDa)	Ð ^d^	1,4-Content ^e^ (%)	1,4-*Cis* ^f^(%)	T*_g_* ^g^ (°C)
PF	-	0	~100	109	204.1	107	3.8	97.7	94	−76.6
PF-1	CNC	0.5	98	81	200	137	3.9	97.6	N.D.	−76.6
PF-2	CNC	1.5	94	61	191.8	281	4.4	97.5	N.D.	−76.8
PF-3	CNC	3.0	87	44	177.6	1443	3.5	97.4	93.3	−79.4
PF-4	CNC	5.0	82	37	167.4	2622	5.5	97.1	N.D.	N.D.
PF-5	*m*-CNC_150_	0.5	99	91	202	175	4.0	97.5	N.D.	−76.8
PF-6	*m*-CNC_150_	1.5	96	68	195.9	267	4.1	97.3	N.D.	−76.1
PF-7	*m*-CNC_150_	3.0	90	51	183.7	1304	3.4	97.6	93.0	−76.1
PF-8	*m*-CNC_150_	5.0	87	44	177.6	2296	4.8	97.4	N.D.	−76.8

All reactions were performed in cyclohexane at 70 °C. The catalytic system used was Nd V_3_/DIBAH/Me_2_SiCl_2_ with 1/20/1 molar ratio. *Trans*-β-farnesene reactions were carried out using a monomer/Nd molar ratio of 500/1 and the total reaction time was 30 min.^a^ Final reaction yield percentage calculated by gravimetry. ^b^ The apparent first-order rate constant k (L * mol^−1^ * min^−1^) was calculated considering the kinetic law d[M]/dt = K[NdV_3_][M] and from plots −Ln(1 − x) = f(*t*), where x is the conversion. ^c^ Catalytic activity calculated after 30 min of reaction (Kgpolymer/molNd·h). ^d^ Determined by SEC. ^e^ Calculated from the ^1^H NMR (see Figure S4). ^f^ Calculated from the ^13^C NMR (see Figure S5). ^g^ Determined by DSC.

**Table 3:** Molecular weight characteristics of a new set of PF’s with CNC and *m*-CNC.

Sample	Filler	wt%	$M_{n}$ (kDa)	Ð
PF	-	0	28	3.8
PF-1	CNC	0.5	35	3.9
PF-9	CNC	1.5	39	4.3
PF-10	CNC	3.0	19	4.8
PF-5	*m*-CNC_150_	0.5	43	4.0
PF-6	*m*-CNC_150_	1.5	65	4.1
PF-11	*m*-CNC_150_	3.0	30	4.8

All reactions were performed in cyclohexane at 70 °C. *Trans*-β-farnesene reactions PF, PF-1, PF-5, and PF-6 were carried out using a monomer/Nd molar ratio of 500/1. The PF-9, PF-10, and PF-11 were carried out using a monomer/Nd molar of 220/1, 250/1, and 480/1 (respectively) to obtain PFs with a similar molecular weight.

## Data Availability

The data presented in this study are available on request from the corresponding author.

## Supplementary Materials

The following are available online at https://www.mdpi.com/article/10.3390/polym13162810/s1, Figure S1: XPS survey of modified and unmodified cellulose nanocrystals, Figure S2: Micrographs of the presence of an agglomerate by AFM height mode, Figure S3: Molecular weight distributions of polyfarnese, Figure S4: ^1^H NMR spectra of the reference polyfarnesene (PF), Figure S5: ^13^C NMR spectra of the reference polyfarnesene (PF), Table S1: Atomic composition of nanocrystals calculated from XPS.
