# Supplementary material for: Fully Bio-Based Elastomer Nanocomposites Comprising Polyfarnesene Reinforced with Plasma-Modified Cellulose Nanocrystals
Source: Polymers (Basel). 2021 Aug 21;13(16):2810. doi: 10.3390/polym13162810 (PMC8401937; doi:10.3390/polym13162810)
Supplement: Supplementary file 1 [file polymers-13-02810-s001.zip › polymers-1324664-supplementary.pdf]

Supporting Information

Fully bio-based elastomer nanocomposites comprising polyfarnesene reinforced with plasma-modified cellulose nanocrystals.

Ilse Magaña[a], Dimitrios Geourgouvelas[b], Rishab Handa[c], María Guadalupe Neira Velázquez[a], Héctor Ricardo López González[a], Francisco Javier Enríquez-Medrano[a], Ramón Díaz de León[a*] and Luis Valencia[d*]

[a]Research Center for Applied Chemistry, *Blvd Enrique Reyna 140, San José de los Cerritos, 25294, Saltillo, Coahuila, Mexico. Email: ramon.diazdeleon@ciqa.edu.mx*
[b]*Division of Materials and Environmental Chemistry, Stockholm University, Frescativägen 8, 10691, Stockholm, Sweden.*
[c]*Experimental Physics, Saarland University, 66123, Saarbrücken, Germany.*
[d]*Biofiber Tech Sweden AB, Norrsken Hourse, Birger Jarlsgatan 57 C, SE11356, Stockholm. Email: luisalex_val@hotmail.com*

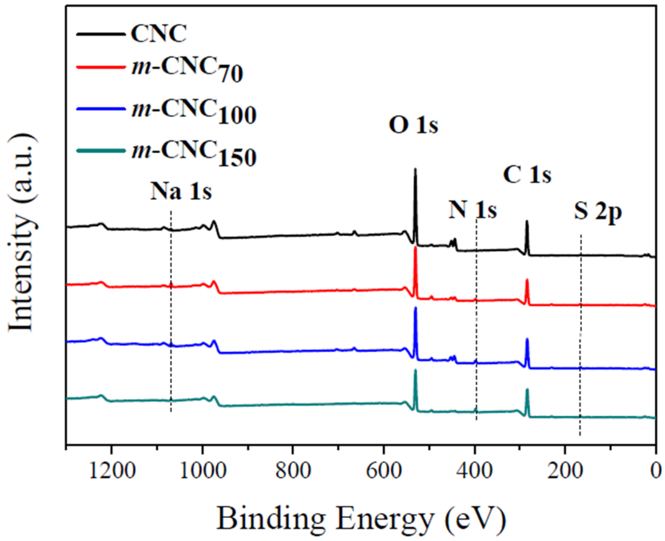

Figure S1. XPS survey of modified and unmodified cellulose nanocrystals.

Table S1. Atomic composition of nanocrystals calculated from XPS.

Sample	O%	C%	C/O	S%	Na%	N%
**CNC**	67.05	31.75	0.47	0.42	0.78	-
***m*-CNC$_{70}$**	62.57	34.28	0.54	0.4	0.99	1.76
***m*-CNC$_{100}$**	59.02	36.94	0.62	0.72	0.76	2.55
***m*-CNC$_{150}$**	53.02	41.75	0.78	0.72	0.36	4.16

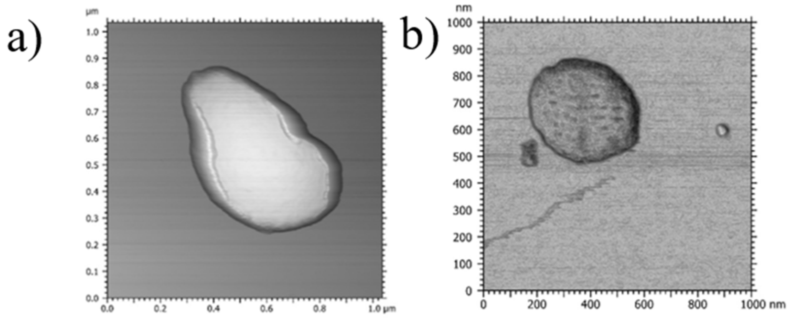

Figure S2 Micrographs of the presence of an agglomerate by AFM height mode.

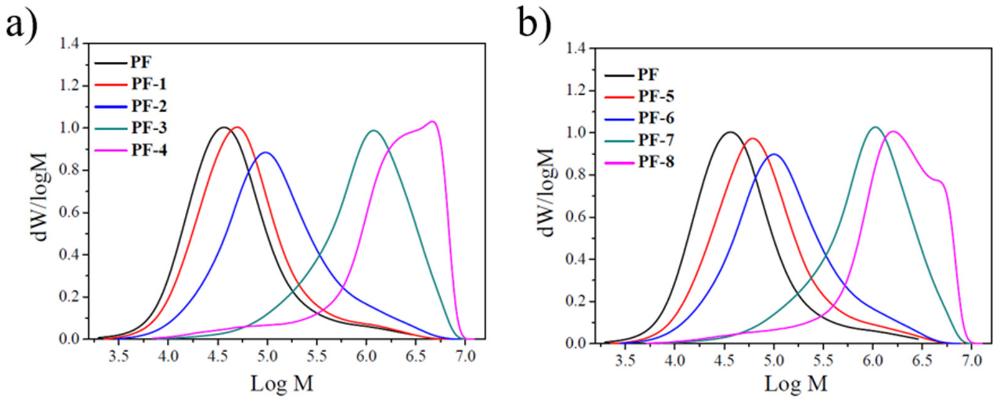

Figure S3. Molecular weight distributions of polyfarnese.

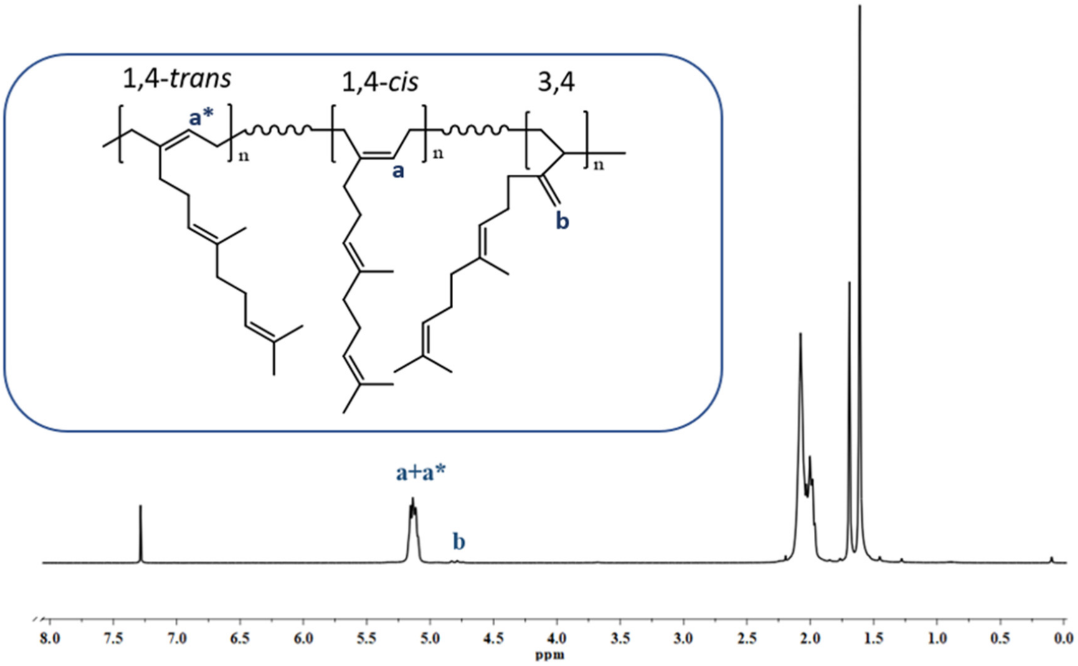

Figure S4. ^{1}H NMR spectra of the reference polyfarnesene (PF).

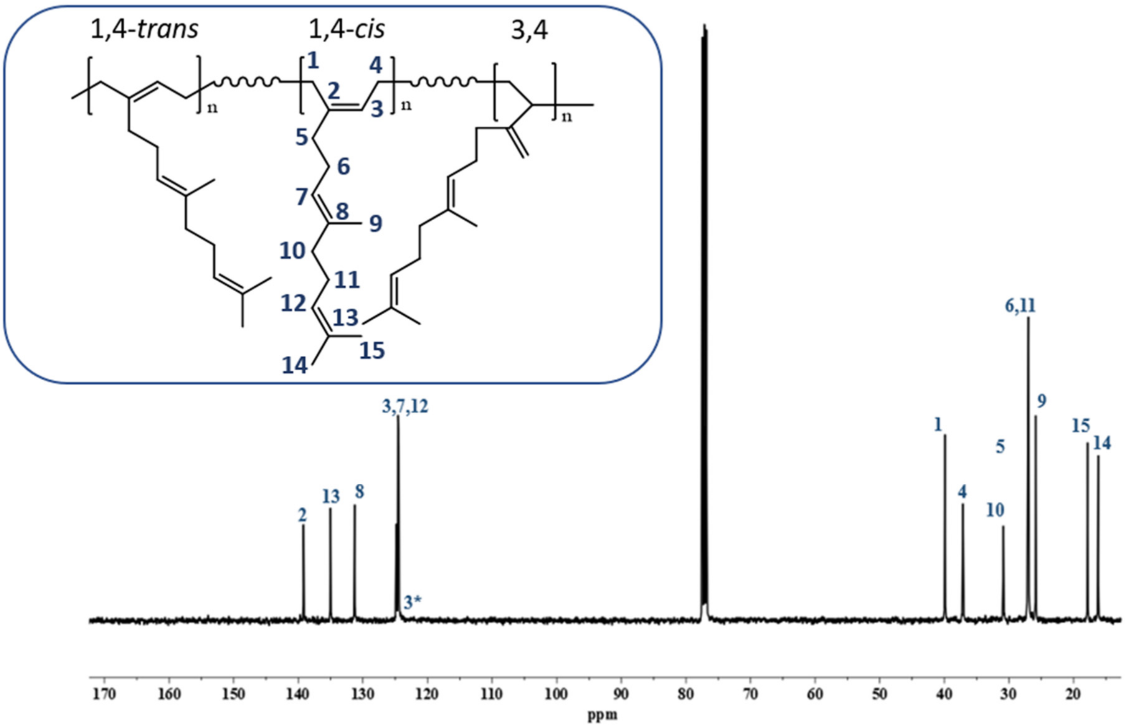

Figure S5. ^{13}C NMR spectra of the reference polyfarnesene (PF).
